# Recent Progress on the Gold-Free Integration of Ternary III–As Antimonide Nanowires Directly on Silicon

**DOI:** 10.3390/nano10102064

**Published:** 2020-10-19

**Authors:** Ezekiel Anyebe Anyebe

**Affiliations:** School of Engineering, Cardiff University, Cardiff CF24 3AA, UK; anyebee@cardiff.ac.uk

**Keywords:** III–As–Sb, nanowires, silicon, gold-free, InAsSb, GaAsSb

## Abstract

During the last few years, there has been renewed interest in the monolithic integration of gold-free, Ternary III–As Antimonide (III–As–Sb) compound semiconductor materials on complementary metal-oxide-semiconductor (CMOS)—compatible silicon substrate to exploit its scalability, and relative abundance in high-performance and cost-effective integrated circuits based on the well-established technology. Ternary III–As–Sb nanowires (NWs) hold enormous promise for the fabrication of high-performance optoelectronic nanodevices with tunable bandgap. However, the direct epitaxial growth of gold-free ternary III–As–Sb NWs on silicon is extremely challenging, due to the surfactant effect of Sb. This review highlights the recent progress towards the monolithic integration of III–As–Sb NWs on Si. First, a comprehensive and in-depth review of recent progress made in the gold-free growth of III–As–Sb NWs directly on Si is explicated, followed by a detailed description of the root cause of Sb surfactant effect and its influence on the morphology and structural properties of Au-free ternary III–As–Sb NWs. Then, the various strategies that have been successfully deployed for mitigating the Sb surfactant effect for enhanced Sb incorporation are highlighted. Finally, recent advances made in the development of CMOS compatible, Ternary III–As–Sb NWs based, high-performance optoelectronic devices are elucidated.

## 1. Introduction

### 1.1. Ternary III–As–Sb Nanowires

In recent years, ternary III−As Antimonide (III–As–Sb) materials have increasingly attracted enormous interest as potential building blocks for next-generation optoelectronics. Among these, the InAsSb and GaAsSb alloys, have particularly attracted increasing attention, due to their intriguing and promising properties. The InAsSb material possesses a tunable bandgap, enabling bandgap engineering for controlled characteristics and device application with its direct bandgap having the smallest energy among all the entire III–V semiconductors (145 meV for *x* =  0.63 at 0 K) [1,2]. It’s potential to extend the detection wavelength limit of InAs (3.8 µm) to the long-wavelength infrared range (LWIR) (8–12 µm) [3] has attracted enormous interest for diverse applications, including photodetectors as a potential replacement to existing HgCdTe-based infrared LWIR detectors which is plagued by concerns of toxicity [4], surface instability, high growth, and processing cost, as well as non-uniformity [5]. In addition, Sb incorporation in InAs opens up opportunities for the investigation of important material-related properties, such as spin-orbit coupling and quantum confinement [2], as well as increases the minority carrier lifetime and mobility [6], enabling highly efficient field-effect transistors. The InAsSb material also offers several advantages, including low electron effective mass, high mobility at room temperature, and reduced auger recombination rate (auger coefficient as low as 10^−7^ cm^6^/s) [7]. On the other hand, the ternary GaAsSb alloy possesses a wavelength covering a highly important range from the near IR (0.87 µm for GaAs) to the mid-IR (1.7 µm for GaSb) region, which holds enormous potential for applications in optoelectronic devices, including optical telecommunications, quantum information science, solar cells, and infrared photodetectors [8,9].

During the last few years, there has been renewed enthusiasm in the monolithic integration of Ternary III–As–Sb compound semiconductors on complementary metal-oxide-semiconductor (CMOS)—compatible silicon substrate to exploit the fascinating properties of the former, including direct bandgap, exceptional optical properties, and high carrier mobility, as well as the scalability, availability and high-quality of silicon to enable application in high-performance, cost-effective devices and integrated circuits based on the well-established Si technology. However, the epitaxial growth of III–V semiconductors on Si is challenging owing to large differences in lattice mismatch, thermal expansion coefficient, and crystal structure (whereas III–Vs have a zinc blende or wurtzite structure, Si has a covalent diamond structure) [10], as well as the polar/nonpolar nature of semiconductors/Si substrate [11] which often results in strain-induced defects and degradation of material quality [12]. One-dimensional nanowires (NWs) provide the panacea for the integration of these materials directly on highly mismatched substrates. The small footprint of NWs in contact with the substrate could be exploited to evade the strict lattice matching requirements for the growth III–V semiconductor thin-film on highly lattice-mismatched substrates, due to elastic strain relaxation enabling the monolithic integration of NWs on silicon. The heteroepitaxial growth of high-quality III–V–Sb NWs directly on Si substrates would undoubtedly open the flood gates for the experimental study of the band structure, carrier transport, and other important fundamental properties of III–As–Sb/Si heterojunctions which are not readily available in conventional thin-film structures [13,14]. In addition, it would enable the independent control of the NWs geometry for optimal device functionality [15]. However, the nucleation and growth of III–As–Sb NWs directly on substrates is extremely challenging [16] and requires strict compliance with contact angle requirements [17,18] owing to the surfactant effect of Sb on the substrate. As an alternative, previous ground-breaking research that provided great insight into the growth of Sb-based NWs, utilized NWs stems, such as InAs [12,19] and InP [2], to facilitate vertical NW directionality. More so, the growth of GaAsSb ternary alloy is particularly made more difficult, due to the existence of a large miscibility gap, 0.25 < *x* < 0.7 [20]. Furthermore, Au (which is the most commonly used catalyst) is incompatible with CMOS processing [21] and could potentially result in the unintentional incorporation of impurities which adversely degrade NWs properties, a phenomenon which has mitigated its potential for use in the monolithic integration of semiconductor NWs with silicon.

This paper provides a detailed review of CMOS compatible, Au-free growth, and optoelectronic applications of Ternary III–As–Sb NWs directly on Si, specifically InAsSb and GaAsSb NWs. We do not attempt to explicitly cover all III–Sb NWs grown on III–V substrates, NWs stems and axial heterostructures, etc., which are inclusive of Au-catalyzed NWs. Broad and more general reviews encompassing the growth of such Sb-based NWs can be found elsewhere [18,22,23,24,25]. First, a comprehensive review of recent progress made in the Au-free growth of InAsSb and GaAsSb NWs directly on Si is presented, followed by a detailed description of the Sb surfactant effect and its influence on both NWs morphology and structure of Ternary III–As–Sb NWs. Then, the various strategies that have been successfully deployed for the suppression of the Sb surfactant effect are highlighted. Finally, recent advances made in the development of CMOS compatible, Ternary III–As–Sb NWs-based devices are explicated.

### 1.2. Au-Free Nanowires Growth Strategies

Various strategies have been employed for the Au-free growth of Ternary III–V–Sb NWs on Si including the self-catalyzed and selective area growth (SAG), which are discussed in the following section.

#### 1.2.1. Self-Catalyzed Growth

The self-catalyzed growth (SCG) technique involves the use of a low melting group III element (such as Ga or In), which is one of the constituent elements of the NW material, as the seed catalyst for facilitating NWs growth [26]. This circumvents the problem of unwanted introduction of impurities usually associated with the use of gold, which is the most used catalyst for NWs growth and potentially provides a safe route for the growth of high-purity NWs compatible with the CMOS technology. For group III–V NWs, the group-III elements are pre-deposited on the substrate to nucleate and drive NWs growth. The SCG of both binary InAs [27,28], GaAs [29], and GaSb [30] and ternary InAsSb [31,32], GaAsSb [33] NWs have been reported. The self-catalyzed growth technique is sometimes referred to as self-assisted [34,35] or self-seeded [36] growth, particularly when the group III element is spontaneously formed on the substrate during NWs growth and not intentionally introduced into the growth chamber prior to commencement of growth. Although, the SCG of NWs is challenged by the control of NWs density and diameter. It has been recently demonstrated that the droplet epitaxy could be used to potentially tune the diameter and density of InAs [28] and GaAs [37] NWs. Thus, unraveling a controllable and cost-effective means of fabricating functional monolithic hybrid NWs structures on silicon.

#### 1.2.2. Selective Area Growth

The Selective-area growth (SAG) of NWs is a positioned controlled epitaxial technique that involves the deposition of a dielectric mask (such as SiO_2_) on (111) oriented substrate followed by the lithographic etching of a well-defined array of holes on the mask either intentionally by electron beam lithography or interaction with group III adatoms [38] to expose the substrate surface for direct fabrication of NWs. Consequently, the predefined holes facilitate NWs nucleation and growth such that the position and size of the NWs are dictated by the holes while at the same time providing favorable conditions for droplet formation [39]. It’s worthy to note that the oxide sidewall possibly serves as a scaffold for limiting adatom mobility to guide the one-dimensional growth [40]. SiO_2_ islands are also believed to catalyze NWs growth, especially in the absence of any foreign catalyst [41]. SAG of InAs [41,42], GaAs [38,43], InAsSb [44,45], and GaAsSb [46,47] have been reported in literature.

### 1.3. Nanowire Growth Techniques

Different growth techniques are currently being utilized for the growth of compound semiconductor NWs on Si substrates, including chemical vapor deposition (CVD), metal-organic chemical vapor deposition (MOCVD), molecular beam epitaxy (MBE), and chemical beam epitaxy (CBE). Chemical Vapor Deposition (CVD) is a chemical vapor phase deposition method in which volatile precursors are decomposed on a heated substrate surface via a chemical reaction resulting in the formation of single or polycrystalline thin films. The technique was first developed in the 1880s, however, it was not until the 1960s that it was used for the fabrication of semiconductor. MOCVD, otherwise known as Metalorganic vapor-phase epitaxy (MOVPE) or organometallic vapor-phase epitaxy (OMVPE), is a specialized form of CVD involving the use of at least one metal-organic (MO) precursor. It employs a mixture of Group III metal-organic and Group V hydride precursors in a carrier gas (H_2_, N_2_, or a mixture of both) for the growth of III−V compound semiconductors.

MBE is an advanced ultra-high (UHV) vacuum epitaxial technique utilized for the growth of compound semiconductor materials by the reaction of one or more thermal molecular or atomic beams of the constituent elements with a heated crystalline surface. The MBE growth process involves the evaporation or sublimation, condensation, and impingement of localized molecules or atomic beams in a UHV environment from ultrapure elements, such as In, Ga, and Al, contained in crucibles confined in effusion or Knudsen cells on structurally suitable substrates heated to the required growth temperature. The intensity of the beam is dictated by the temperature of the solid source in the effusion cells, while the substrate temperature provides sufficient thermal energy to the arriving atoms to migrate over the surface to lattice sites and eventually incorporate into the growing film. The UHV environment creates the needed ambiance for a near collision-free transmission of the beams while minimizing the contamination of the growing surface to ensure the growth of high-purity semiconductors. MBE exhibit several advances, such as low growth rate (typically 1 μmhr^−1^), which (combined with the UHV permit precise real-time composition and monolayer thickness control) result in the growth of high-quality crystals with smooth surfaces while enabling independent control of growth and precursor temperatures. MBE is equipped with in-situ growth monitoring by sophisticated diagnostic tools, including Reflection High-energy Electron Diffraction (RHEED), for direct measurements of the surface structure of the growing layer and Auger Electron Spectroscopy for examining the surface chemical composition of the substrate or growing epilayer [48]. However, depending on the nature of the source, the term MBE is strictly applied when a solid source is used, whereas it is gas source MBE (GS-MBE) when gaseous sources are used. CBE (otherwise called MO-MBE) vary from MBE in the sense that metal-organic compound sources combine the characteristic beam of MBE while using MO vapor source as in MOCVD. The growth of crystals by MBE is a physical process that contrasts the chemical reaction of MOCVD. Given the significant difference in the deposition process between MBE and MOCVD, including growth rates and mechanisms, the influence of Sb incorporation on NWs growth will be discussed separately in the next session.

## 2. Growth of Au-Free Ternary III–As–Sb Nanowires Directly on Silicon

To monolithically integrate highly efficient Ternary III–V–Sb NWs-based devices (such as infrared detectors) with the Si-based read-out circuit [49], NWs have to be directly grown on the Si substrate. During the last few years, significant progress has been made on the Au-free growth of ternary III–V–Sb NWs directly on silicon using various growth methods, including MBE, MOCVD, MOVPE, and CBE. Here, we summarize the recent progress made in the growth of InAsSb and GaAsSb NWs directly on silicon.

### 2.1. InAsSb Nanowires Growth Directly on Silicon

**Figure 1:**
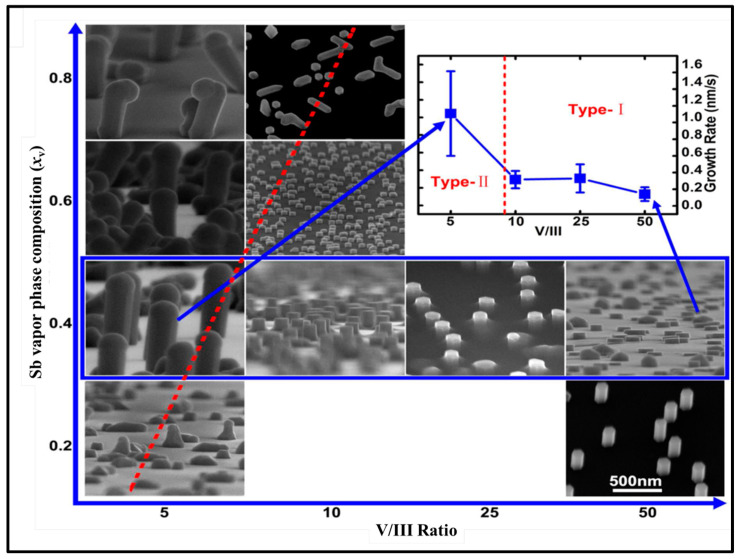
Images of InAsSb NWs as a function of *x*_v_ and V/III ratio. The inset in the top right shows the variation of growth rate with the V/III ratio for *x*_v_ = 0.4 (as highlighted by the blue rectangular frame). The solid line is used for eye guide, indicating the changing trend of growth rate. The red dashed line in the figure indicates the transition boundary between vapor–solid and vapor–liquid–solid growth modes. Reproduced with permission from [54]; Copyright 2015, American Chemical Society.

There is an extensive body of literature on the growth of Au-catalyzed heterostructured InAs/InAsSb [50,51] and InAsSb NWs on InAs(111)B substrate [12]; however, the first successful growth of InAsSb NWs directly on Si (111) was demonstrated by Du et al. [36] in 2013, via the self-seeded growth mechanism by MOVPE. The Sb content was found to have a significant effect on the morphology and crystal quality of the NWs. The length of NWs decreased from (0.15–0.60) μm to (0.39–0.07) μm while the diameter increased from (27–65) nm to (434–698) nm with increasing Sb vapor phase composition (*x*_v_) from 0 to 0.8 [where *x*_v_ is the ratio of Sb to the combined (As and Sb) group-V material flux]. Short and thick flat pillars were obtained after 2 min of growth at the highest Sb composition (*x*_sb_) of 43 at.%. This implies an increase in *x*_v_ translates to an enhancement of radial growth with suppression of axial growth. In 2014, our research group [52] also realized the growth of InAsSb NWs on bare Si by MBE via an In-assisted nucleation without using NWs stems. It was observed that the geometry of the NWs was modified by increasing *x*_v_ with an almost doubling of the lateral dimension and a corresponding suppression in NWs axial growth, which is similar to the earlier observation by Du et al. [36]. Several important milestones were achieved in 2015, as there was increased research on the growth of InAsSb directly on Si. Motivated by the observed influence of Sb addition to the geometry of InAsSb NWs, our research group investigated the surfactant effect of Sb addition to the MBE growth of NWs [32] and Sb induced phase control [53]. It was confirmed that trace Sb flux has the potential of tuning the geometry of InAsSb NWs by promoting lateral growth while suppressing axial growth. The observed behavior is attributed to the surfactant effect of Sb, which results in modifications to the kinetic and thermodynamic processes. A thermodynamic mechanism that accounts for Sb segregation in InAsSb NWs was thus elucidated, unraveling a new route towards precisely controlled NW morphology by the addition of Sb (more discussion on this to follow in subsequent sections). Another important step towards a better understanding of the growth of this ternary material was achieved in 2015 when Du et al. [54] fabricated InAsSb NWs on Si (111) substrate by MOVPE as a function of various growth conditions, including V/III ratio, group Sb flow rate fraction, and temperature. They found that InAsSb NWs can display both vapor–liquid–solid (VLS) and vapor–solid (VS) growth mechanism depending on the growth parameters. At low V/III ratio and relatively high Sb flow rate fraction, the VLS growth mechanism is dominant, whereas, at high V/III ratio and relatively low Sb flow rate fraction, NWs were grown via the VS mechanism. There were marked differences in morphology, Sb content, growth direction, and crystal quality of as-grown NWs depending on the growth mechanism, as depicted in Figure 1. The authors observed that the VS grown NWs exhibited uniform growth direction and diameter, with a hexagonal cross section. In addition, the NWs tips were flat with no droplets present, suggesting a catalyst-free growth mechanism with uniform Sb distribution, which is beneficial for device integration of NWs array. Conversely, the VLS grown NWs are almost kink-free and display multiple orientations. The NW top terminates with a hemispherical In–Sb alloy droplet indicative of a catalyst-assisted growth mechanism with significant variation in Sb distribution and display of a pure crystal phase, which was mainly attributed to the In-rich droplets that are advantageous for the development of single NW devices. This work demonstrates that the In-rich droplet of the Au-free growth could potentially improve the crystal quality of 〈111〉-oriented NWs without the introduction of impurities. The growth temperature regime for realizing InAsSb NWs was identified to be in the range of 450–510 °C at an *x*_v_ of 0.2. In addition, the authors observed an inverse relationship between the lateral growth rate of the NWs and growth temperature, with higher growth temperatures favoring axial NWs growth.

In 2020, Wen et al. [55] investigated the influence of essential growth parameters of growth temperature, indium flux, and substrate type on the growth of silver-assisted InAs_1−_*_x_*Sb*_x_* NWs directly grown on Si (111) substrates by MBE. The authors observed the growth of vertically aligned InAs_1−_*_x_*Sb*_x_* NWs directly on Si (111) substrates is extremely difficult; however, the use of relatively high growth temperature and low indium fluxes promotes the growth of vertically aligned NWs with a corresponding suppression in random growth of NWs. On the other hand, the growth of non-[111]-orientation NWs, NWs tapering, and number density was found to increase with increasing In-flux, due to higher indium content resulting in increased segregation of the silver-indium catalyst on the top of the NW during growth. A further increase in Indium composition and catalyst segregation results in a gradual decrease in catalyst diameter, leading to enhanced tapering, since NWs diameter is highly dependent on the size of the catalyst alloy. There was no obvious variation in the morphology and growth direction of the InAs0.85Sb0.15 NWs grown on various Si (111), Si (110), and Si (100) substrates, which were all characterized by the growth of non-[111]-oriented NWs and attributed to the formation of an ultrathin oxide layer on the silicon surface prior to loading into the MBE chamber.

### 2.2. GaAsSb Nanowires Growth Directly on Silicon

**Figure 2:**
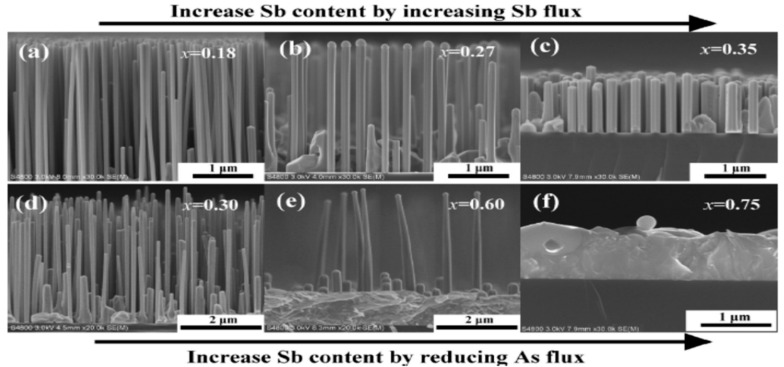
Side-view SEM images of the Ga self-catalyzed GaAs_1__−*x*_Sb*_x_* nanowires grown on Si (111) substrates by molecular beam epitaxy (MBE). (**a**–**c**) GaAs_1__−*x*_Sb*_x_* nanowires were obtained by increasing Sb flux, and the corresponding *x*_sb_ are 0.18, 0.27, and 0.35, respectively. (**d**–**f**) GaAs_1__−*x*_Sb*_x_* nanowires were obtained by reducing As flux, and the corresponding *x*_sb_ are 0.30, 0.60, and 0.75, respectively. Reproduced with permission from [60]; Copyright 2017, American Chemical Society.

Building on the pioneering Au-assisted growth of GaAsSb NWs on GaAs(111)B by Dheeraj et al. [56] in 2008, previous Au-free GaAsSb NWs growth on Si were limited to heterostructures [57,58]. To the best of our knowledge, the first reported Au-free growth of GaAsSb NWs directly on Si was reported in 2013 by Alarcón-Lladó et al. [59], who systematically investigated the optical properties of MBE grown NWs for Sb contents from 0 to 44 at.%, as determined by EDX and provided a reference for the study of ternary NWs alloys while demonstrating the high-quality of gold-free ternary antimonide NWs directly grown on silicon. This report was followed in 2014 by the selective area, MBE growth of Pure zinc blende GaAsSb NWs by the same group [47]. The authors unambiguously demonstrated that the NWs are completely twin-free down to the first bilayer with three-dimensional composition evolution. This work opened the way for the integration of novel infrared nanodevices directly on the cost-effective Si platform. Li et al. [60] systematically investigated Ga-catalyzed, MBE growth of GaAsSb NWs by tuning the Sb and As fluxes achieve near full-composition-range. By tuning the Sb flux from 4.50 × 10^−7^ to 1.43 × 10^−6^ Torr the Sb content increased from 0.18 to 0.35 (Figure 2a–c), with almost no NWs growth, realized on the Si substrates for *x* > 0.45 with limited growth of short and thick NWs. However, by increasing the Sb content from *x* = 0.30 to 0.60, both the density and axial growth rate of the GaAsSb NWs were decreased while the diameter increased, and No NWs growth was realized for a high Sb content of 0.75 at.% (Figure 2f) which was rather dominated by the growth of a two-dimensional film.

A comprehensive study of the effect of Sb incorporation on the composition modulation, structural and optical properties of self-assisted GaAsSb NWs on (111) Si substrate was conducted by Ahmad et al. [61] in 2017 using MBE. The NWs exhibited a pure zinc blende crystal structure, largely free of any planar defects with inverse dependence of NWs density as a function of Sb flux, which was associated with the surfactant effect of Sb and droplet size-dependent Gibbs–Thomson effects [62]. EDX extracted Sb composition in as-grown GaAsSb axial NW varied from 2.8–16 at.%; however, higher Sb incorporation enhanced radial NWs growth with a concomitant reduction in the axial growth rate was observed. The authors revealed that thinner NWs with low Sb composition presents inhomogeneous Sb composition distribution radially with a depleted Sb surface region, whereas, NWs with larger NWs and higher Sb composition (16 at.%) display a more uniform Sb compositional distribution radially leading to type-I optical transitions. This variation in Sb distribution was attributed to differences in growth mechanism. In the same year, the influence of group V/III beam equivalent (BEP) ratios and substrate temperature on the density and chemical composition of self-catalyzed, MBE grown GaAsSb NWs on p-type Si (111) substrate was systematically Investigated [33]. A two-step growth temperature sequence of initiating growth at a relatively higher temperature and then continuing the growth at a lower temperature was shown to be a promising technique for realizing a high-density of NWs despite higher Sb compositions.

**Figure 3:**
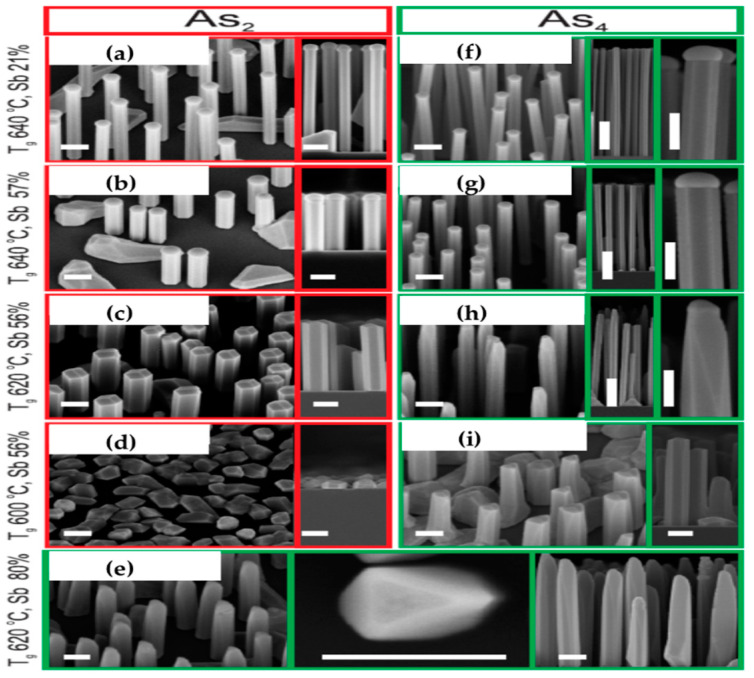
SEM images of GaAsSb NWs grown on Si (111) substrates. The scale bars are 200 nm, except in the lower magnification insets of sample h, they are 1 μm. Sb% refer to Sb flux%. The samples with red outlines (**a**–**d**) have been grown with As_2_ while those with green outline (**e**–**i**) were grown with As_4_. Reproduced with permission from [63]; Copyright 2020, IOP publishing Ltd.

To engineer the optical and electrical properties of semiconductor NWs for various optoelectronic applications, it is important to precisely control essential growth parameters. In 2020, the influence of As_2_ and As_4_ species on the growth of self-catalyzed GaAsSb NWs was investigated by Koivusalo et al. [63] by MBE via a droplet epitaxy growth technique. The authors demonstrated that a careful selection of As species is critical for tuning the NW dimensions and the incorporation mechanism of Sb. It was shown that As_4_ is the preferred candidate of choice when axial NWs growth is essential, whereas As_2_ is the best alternative when radial growth is required. As can be seen from Figure 3, the NWs grown using As_2_ under identical growth conditions as their As_4_ counterparts have a relatively larger diameter, whereas those grown with As_4_ are longer in length. As_4_ mitigates Sb induced suppression of NW aspect ratio for enhanced axial growth while favoring the extension of the axial growth parameter window, to enable the growth of GaAsSb NWs with high (47%) Sb composition. The observed As_4_ induced enhanced axial growth is associated with the As incorporation kinetics. As_4_ has a lower sticking coefficient compared to As_2_ on the common sidewall surface of self-catalyzed GaAs NWs. As a result, there is less efficiency As incorporation on the sidewall from As_4_ compared to As_2_ during VS growth, which implies an increase in local V/III ratio in the vapor phase with increased droplet supersaturation and axial NW growth rate. On the other hand, As_2_ promotes sidewall nucleation for enhanced radial growth, due to its relatively high sticking coefficient.

## 3. Surfactant Effect of Sb

The phenomenon whereby Sb atom tends to float or segregate at the surface of a growing layer rather than being incorporated is known as the Sb surfactant effect and associated with the low volatility of the relatively heavy Sb atom (atomic number of 51) [18,64]. Sb segregation is closely linked to the bond energetics of the constituent materials, particularly the difference in bond strength between the group III–V elements. For instance, while the binary In−As is tightly bound (48.0 kcal/mol^–1^) [65,66], the Sb–Sb bond is less tightly bound (30.2 kcal·mol^–1^) [67,68], and consequently, the Sb atom is more likely to be expelled from the ternary InAsSb material to form a floating layer [69,70]. Similarly, for the GaAsSb alloy, the Ga–As has a strong bonding (50.1 kcal·mol^–1^) [66]; hence, there is a ready preference for the Sb atom to be expelled without being incorporated. According to Treglia et al. [71], the three key drivers of equilibrium surface segregation includes [72] (I) differences in surface energy between Sb and the binary alloys (including InAs or GaAs) (II) differences in atomic size [73] and (III) the propensity for phase separation. A critical analysis of the ternary In–As–Sb and Ga–As–Sb systems reveals: Sb has relatively smaller surface energy compared to both As and Ga [74,75], its addition would lead to a decrease in surface energy of the binary alloy (InAs or GaAs) in favor of increased Sb segregation. Furthermore, Sb has a larger atomic size; its addition would increase the alloy lattice parameter, leading to a decrease in steric effect. Finally, Sb/As is particularly known to exhibit energies intermixing [76]. For these reasons, the ternary III–V Sb compound has a strong tendency to segregate and display its surfactant property with implications on NWs morphology and crystal structure.

### 3.1. Influence of Sb Surfactant on MBE Grown III–As–Sb Nanowires

In this section, the influence of Sb in addition to the growth of III–As–Sb NWs will be discussed, starting with InAsSb and followed by GaAsSb NWs. Intriguingly, almost all the available studies of IIII–As–Sb NWs on Si reported to date were synthesized by the MBE growth technique.

#### 3.1.1. Influence of Sb Surfactant on Nanowire Morphology

**Figure 4:**
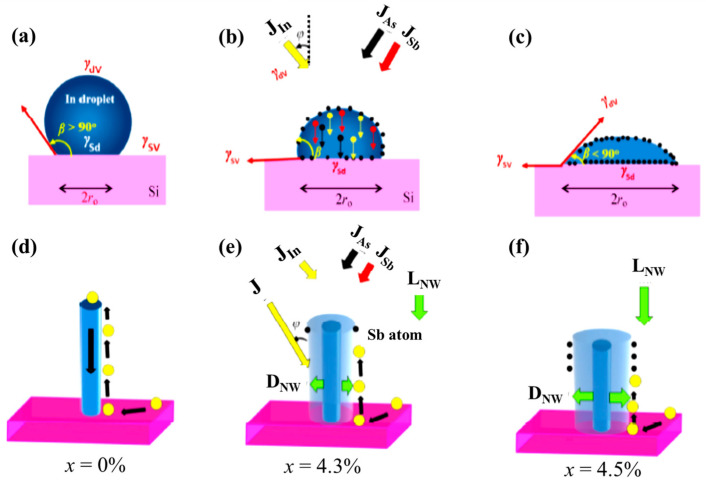
Schematic illustration of the effect of Sb surface segregation on the geometry of indium droplet (**a**–**c**) and suppression of InAs NWs axial growth (**d**–**f**). *J*I_n_, *J*_Sb_, and *J*_As_ denote the In, Sb, and As flux, respectively. The balance of forces acting on a droplet placed on a substrate are also shown with *γ*_dv_, *γ*_sd_, *γ*_sv_ representing the surfaces energies at the droplet–vapor, solid–droplet, and solid–vapor interface, respectively, and *β* is the contact angle between droplet and substrate. Note that the NW dimensions are not drawn to scale and do not represent the extent of Sb-induced modification to the NW geometry. Reproduced with permission from [32]. Copyright 2014, Springer Nature.

The geometry of InAsSb NWs is significantly modified by the presence of trace Sb content resulting in enhanced lateral growth with concomitant suppression of axial growth. Recent studies have shown that this morphological anomaly is independent of the duration of growth. Anyebe et al. [32,52] demonstrated that the geometry of InAsSb NWs deposited for both short and long growth durations by MBE were significantly changed by the presence of Sb. Whereas, the reference InAs were ~65 nm wide and ~900 nm long, the InAsSb NWs grown for 20 min with trace Sb composition (4.3 and 4.5 at.%), had their diameters increased to 109 and 115 nm while their length was reduced to ~870 and 825 nm, respectively, as shown in Table 1. A similar trend was also observed for the NWs deposited for a long growth time of 120 min. Although, the Sb-free InAs reference was ~77 nm wide and 3.82 μm long, the addition of 10.2 at.% Sb resulted in NWs with a significantly large diameter (~155 nm) and suppressed length (0.7 μm) [32]. A slight increase in Sb content to 14.5 at.% completely suppressed NWs growth resulting in the growth of a 2D film. Sb is known to significantly influence both the nucleation and the growth of III–V–Sb NWs. On NWs nucleation, Sb adsorption and segregation modify the geometry of the nucleation droplet and lowers the interfacial surface free energy at both the droplet–vapor interface and growth front, due to its small surface energy (0.38 J·m^–2^) [74]. Such droplet modifications combined with changes in composition results in a reduction in nucleus contact angle (Figure 4a–c), which potentially contributes to the observable changes in NWs geometry. Furthermore, the observed enhancement in lateral growth and accompanying suppression of axial growth is also attributed to the surfactant effect of Sb, which results in modifications to the kinetic process of NWs growth. The incorporation of Sb and the resultant enlargement in InAsSb NWs diameter is associated with significant modifications to the growth mechanism of the binary InAs NWs. Firstly, Sb induces a site-blocking or “poisoning” effect mitigating the incorporation of In and As precursors into the NWs growth process, which dramatically reduces NW axial growth rate while favoring lateral growth (Figure 4b). Secondly, the adsorption of Sb on the NWs side facets kinetically imposes certain limitations on adatom diffusion from the substrate and sidewalls towards the growth front, reducing the adatom diffusion flux to the NW top and mitigating axial growth (Figure 4d,e). With increased Sb flux and surface segregation, adatom diffusion is further suppressed with increased sidewall nucleation and lateral growth, leading to pronounced suppression of axial growth and enhancement of diameter enlargement. The use of an extremely high Sb flux with high Sb content (14.5%) eventual results in a transition to 2D film.

Sb-induced morphological transformation is not limited to InAsSb NWs and has also been observed in GaAsSb NWs [60,61]. Ren et al. [46] observed that the morphologies of MBE grown GaAsSb NWs are significantly influenced by the Sb flux, whereas, the diameter of the NW increased, the lengths decreased with increasing Sb flux. The GaAsSb NWs with Sb content of (0.18, 0.27, and 0.35) had increasing diameters of (~75, 140, and 175) nm, and decreasing axial growth rates (79, 71, and 22) nm/min, respectively (Table 1). The observed morphological abnormally was attributed to the Sb surfactant effect with Sb floating on top of the growing surface. It is believed that the Sb and As atoms were exchanged at the growing surface such that the surface is floating Sb atoms combined with the Ga atoms adsorbed on the (110) NW side-facet, reducing Ga diffusion on the facets while promoting radial growth. Density functional theory (DFT) calculation was used to explain the increased Ga binding energy to the NWs side facets in the presence of adsorbed Sb promoting the crystallization of NWs on the side facets leading to enhanced radial growth. On the other hand, the anomalous decrease in axial growth rate with increased Sb flux was attributed to a combination of indirect kinetic influence involving the Ga adatom diffusion induced evolution of the catalyst geometry and direct composition modulation. Higher Sb flux results in increased Sb-induced obstruction of Ga adatom diffusion to the catalyst and reduction in the Ga catalyst contact angle [32], which indirectly reduces the As collection efficiency leading to the reduced axial growth rate. This is in addition to the Sb-induced direct composition modulation and decreased supersaturation, which also reduces axial growth rate [46].

#### 3.1.2. Influence of Sb Surfactant on Nanowires Crystal Structure

**Figure 5:**
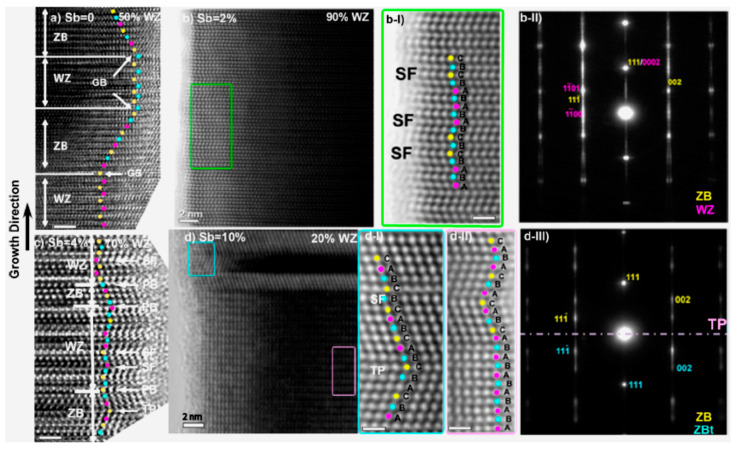
High-resolution TEM (HRTEM) images of InAs_1__−*x*_Sb*_x_*NWs with Sb content of (**a**) 0, (**b**) 2, (**c**) 4, and (**d**) 10%. The InAs NWs (Sb content of 0%) have a ZB dominant structure with WZ fraction of 20%. The addition of Sb with the content of 2% and 4% resulted in WZ predominant phase, while the further increase of Sb content (10%) led to ZB predominant structure. Magnified HRTEM image of the highlighted region of sample (**b**) is shown in (**b-I**) with the corresponding fast Fourier transform (FFT) pattern showing in (**b-II**). Magnified HRTEM images of the highlighted regions of the sample (**d**) are shown in (**d-I** and **d-II**) with the corresponding FFT pattern showing in (**d-III**). These magnified images show the stacking in the structure, revealing ZB and WZ structures and SF and TP. The scale bar is 1 nm. Reproduced with permission from [53]; Copyright 2015, American Chemical Society.

Au-free, binary InAs [13,34,77] and GaAs [78,79] NWs usually display the zincblende (ZB) and wurtzite (WZ) phase mixture. This has mostly been associated with the lower surface energy of the WZ phase in comparison to the corresponding crystalline orientation of the ZB material. Consequently, the WZ phase is more stable in NW structures with high surface to volume ratio [80,81]. Classical nucleation theory has also been used to explain this phenomenon and attributed it to the lower WZ nucleation barrier compared to that of ZB [82,83,84]. For gold-free III–V–Sb NWs to be successfully integrated on CMOS compatible silicon, it is essential that the NWS are completely free of planar defects, including twins, stacking faults (SFs), or polytypism. Impressive experimental demonstrations of Sb-induced transformation of structural defects have recently been demonstrated. In 2015, Zhuang et al. [53] reported for the first time, the complete control of the crystal structure of InAsSb NWs by tuning the Sb composition. Although self-catalyzed InAs NWs usually display a mixture of WZ and ZB phases, the addition of trace Sb (~2–4 at.%) promoted a structural transformation to quasi-pure WZ InAsSb NWs, while a further increase in Sb content to ~10% resulted in a quasi-pure ZB InAsSb NWs, as shown in the high-resolution transmission electron microscopy (HRTEM) image in Figure 5. This was further confirmed by photoluminescence (PL) spectroscopy, which revealed that the type II, quantum well-related emission observed in the InAs NWs PL, due to the coexistence of ZB and WZ phases was conspicuously absent in the InASb NWs which rather showed a clear band edge emission attributed to the quasi-pure WZ structure of the NWs. This confirmed that the crystal structure of the highly defective reference InAs NWs (Sb = 0%) had evolved to a less defective structure with trace Sb addition. Similarly, the SFs density monotonically decreased with increasing Sb concentration, due to the suppression of SFs formation in the NWs. The study provided new insights into the role of Sb addition regarding the effective control of the NW crystal structure. The observed Sb-induced crystal phase transition can be explained using the following nucleation model [82,85,86]:

(1) $$\xi =\frac{\Delta G_{WZ}}{\Delta G_{ZB}}=\;\frac{\Delta {µ}_{LS}\eta^{2}}{\Delta {µ}_{LS}-\Psi_{WZ}}$$

where ξ is the ratio between the WZ (Δ*G*_WZ_) and ZB (Δ*G*_ZB_) nucleation barriers, $\Delta {µ}_{LS}$ is the supersaturation at the liquid-solid interface and $\Psi_{wz}$ is the additional cohesive energy needed for the formation of a WZ layer at the triple-phase line (TPL), η is the ratio between the effective surface energies of the WZ (Γ*_WZ_*) and ZB (Γ*_ZB_*) phase and is given by:

(2) $$\eta \;=\;\frac{\Gamma^{wz}}{\Gamma^{zb}}\;=\;\frac{\left(1-x\right)\Upsilon_{LS}^{1}\;-\;x\Upsilon_{LV}sin\beta \;+\;\tau x\Upsilon_{SV\left(ZB\right)}}{\left(1-x\right)\Upsilon_{LS}^{1}\;-\;x\Upsilon_{LV}sin\beta \;+\;x\Upsilon_{SV\left(ZB\right)}}$$

here, Υ*_LS_*, Υ*_LV_*, and Υ*_SV_* are the interfacial surface energies of the liquid–solid, liquid–vapor, and solid−vapor surface energies, respectively. *Τ* = $\Upsilon_{wz}/\Upsilon_{zb}$ is the ratio of the lateral solid–vapor surface energies of WZ and ZB nuclei, and *x* is the share of the nucleus perimeter that is in contact with the vapor. Lower $\Upsilon_{LV}$ (and by implication higher η) values favor the nucleation of ZB monolayers; otherwise, the WZ structure is preferable. Consequently, a significant increase in $\Upsilon_{LV}$ or decrease in $\Upsilon_{LS}$ would promote a decrease in η in favor of the WZ phase nucleation probability. Consequently, the phase transition from polytypic InAs NWs to WZ with addition of trace Sb (~2–4 at.%) could be attributed to Sb-induced reduction in both the contact angle of the catalyst and the liquid−solid (LS) interfacial energy ($\Upsilon_{LS}$), leading to a reduction in η and lowered WZ nucleation barrier enabling preferential nucleation at the TPL. The increased Sb incorporation (~10 at.%) in the NWs possibly resulted in changes in supersaturation of the group-V elements in the In droplet [56], resulting in the WZ → ZB phase transition.

**Figure 6:**
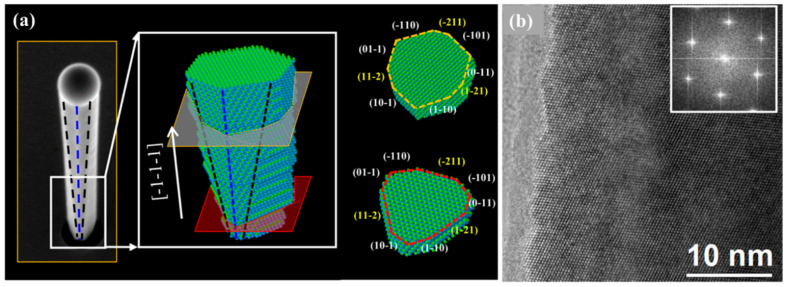
(**a**) High-magnification SEM image of a single NW and associated 3D model revealing the complex faceting composed of {110} and {112} planes. (**b**) High-resolution TEM image was taken along a [0−11] zone axis of a typical NW showing pure twin-free zinc blende crystal structure. Nano faceting is visible on the side of the NW. The inset shows the fast Fourier transform pattern, typical of untwinned zinc blende. Reproduced with permission from [47]; Copyright 2014, American Chemical Society.

**Figure 7:**
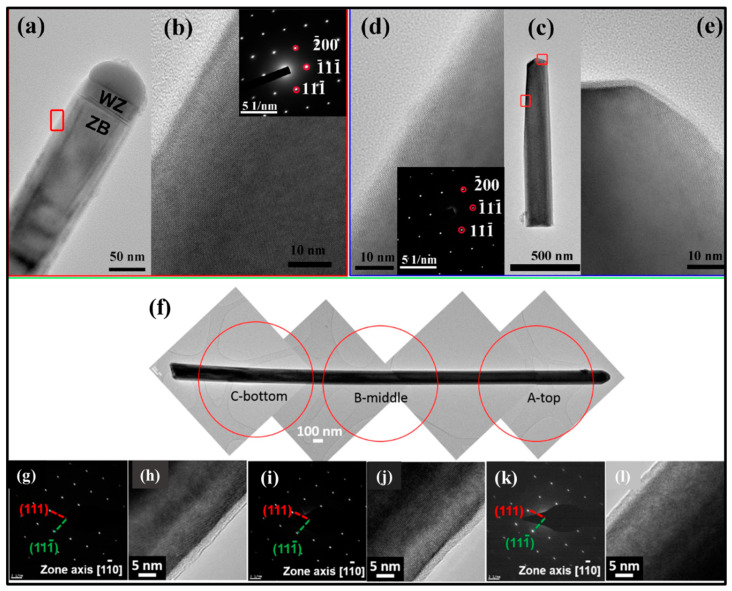
(**a**,**b**) TEM images of a GaAs_1__−*x*_Sb*_x_* nanowire with Sb composition *x*_sb_ of 0.18, (**c**–**e**) TEM images of a GaAs_1__−*x*_Sb*_x_* nanowire with *x*_sb_ of 0.35. Reproduced with permission from [60]; Copyright 2017, American Chemical Society. (**f**) Bright-field TEM image of 16 at.% Sb content GaAsSb nanowire. A, B, and C represent top, middle and bottom locations in the NW, respectively. HRTEM images and corresponding SAED patterns for the (**g**,**h**) bottom, (**i**,**j**) middle and (**k**,**l**) top segments of the nanowire, respectively (zone axis [110]). Reproduced with permission from [61]; Copyright 2017, IOP publishing Ltd.

The addition of Sb in GaAs similarly induces crystal phase modifications in GaAsSb NWs. Conesa-Boj et al. [47] studied the internal crystal structure and three-dimensional composition evolution both at the single NW level and in large ensembles of MBE grown, position-controlled, self-catalyzed GaAsSb NWs on Si (111) with Sb compositions of ~0.17–0.29. By combining reflective high energy electron diffraction (RHEED), TEM, and x-ray diffraction (XRD), the authors unambiguously proved that most of the NWs were pure twin-free ZB crystals down to the first bilayer, which is believed to be the highest level of structural quality in an NW ensemble, over a macroscopic scale. The high-magnification SEM image of Figure 6a shows a single NW and associated 3D model, which reveals the NW exhibit complex faceting composed of six {110} and three {112} facets, with the dominating facet type reversing along the length of the NW. The HRTEM image (Figure 6b) taken along a [0–11] zone axis of a typical NW shows a pure twin-free ZB crystal structure. This novel report of crystal phase perfection, which clearly contrasts the commonly reported defective NWs, demonstrates the enormous potential of Sb for realizing quasi-phase pure III–V–Sb NWs crystals, which is highly promising for novel infrared devices integrated directly on the well-established and cost-effective Si platform. Such a WZ → ZB WZ phase transition is usually ascribed to a lowering of droplet composition [46] and a reduction in supersaturation [46,80,82,85]. Using DFT calculations, Ren et al. [24,46] have shown that Sb incorporation reduces the supersaturation. This reduction in supersaturation is further enhanced by an increase in radial growth, which results in a decrease in both catalyst droplet contact angle and As concentration. This is further promoted by the kinetically inhibited Ga adatom diffusion, which modifies the catalyst geometry. A similar report of Sb induced formation of ZB phase was reported by Li et al. [60]. Detailed HRTEM and SAED (Figure 7a,b) results reveal that the MBE grown, self-catalyzed GaAsSb NW, exhibit a pure ZB crystal structure, except for a very short WZ section underneath the droplet (Sb content = 0.18). The transition from ZB to WZ/stacking faults at the NW tip was attributed to the limited supply of Sb immediately after growth termination favoring nucleation at the TPL. Interestingly, the very short WZ section was only observed in the GaAsSb NWs with low Sb content. The inset of Figure 7b is a SAED of the NW, which is indexed to the face-centered cubic phase of GaAsSb viewed along the [011] axis confirming the ZB phase of the NWs. For higher Sb content of 0.35, the GaAsSb NW completely display a pure ZB structure as evidenced by HRTEM and SAED images (Figure 7c–e), due to the decreased supersaturation of the group-V elements in the Ga droplet in the presence of high Sb flux. Twin free, pure phase ZB GaAsSb NWs crystal has also been demonstrated with 16 at.% Sb content [61] as confirmed by the bright-field TEM (Figure 7f), HR-TEM images and corresponding SAED patterns taken from three different locations (Figure 7g–l) of an NW. Interestingly, despite having a relatively low Sb content, no WZ phase insertions were observed at the NW tip, suggesting that the onset of ZB to WZ transition is also a function of the overall growth conditions employed for NWs growth. The presence of Sb could potentially induce a ZB to WZ transition or otherwise, depending on the balance of highly sensitive parameters, such as surface energy, droplet contact angle, and supersaturation—all of which can be controllably manipulated using the appropriate growth conditions. This suggests a careful selection of appropriate growth precursors, and Sb flux is essential to enable crystal phase engineering of Au-free, III–V–Sb NWs.

### 3.2. Influence of Sb Surfactant on MOCVD Grown III–As–Sb Nanowires

The influence of Sb incorporation on the morphology and structure of III–As–Sb NWs has also been reported for MOCVD grown NWs, which suggests this phenomenon is not associated with the growth technique utilized for the growth. Du et al. [36] reported that Sb content has a significant effect on the morphology of InAsSb NWs. Specifically, they found opposing trends in the axial and radial growth rates of the NWs with increasing vapor phase composition. In addition, the crystal quality of as-grown NWs was improved with a transition from the usual polytypic InAs structure to nearly defect-free NWs, as evidenced by HRTEM and selective area electron diffraction pattern (SAED) analysis, due to the addition of minimal Sb content (*x* = 0.08) which is attributable to modifications in NWs surface energy. However, excessive Sb content (*x* = 0.14) was ineffective in achieving crystal purity as it rather accelerates the formation of defects, which demonstrates that the influence of Sb on phase purity is highly sensitive to the Sb composition combined with other growth conditions.

### 3.3. Strategies for the Suppression of Sb Surfactant Effect

Various strategies have been developed for the suppression of Sb-induced surfactant effect on the morphologies of III–As–Sb NWs, including a decrease in As flux and the use of two growth temperature regimes. Zhuang et al. [87] reported the successful MBE growth of high-quality and optically efficient InAsSb NWs on silicon via an advanced droplet-assisted epitaxy. By optimizing the V/III flux ratio and Sb flux, the Sb surfactant effect was successfully suppressed, leading to the growth of high Sb content of up to 19 at.%, while extending the emission wavelength to 5.1 μm. Similarly, Conesa-Boj et al. [47] reported that Sb incorporation can be significantly increased by reducing the As flux to promote substitution of As with Sb atoms and increase Sb incorporation in GaAsSb NWs. The same strategy was also deployed by Li et al. [60], who successfully increased Sb incorporation from 0.30 to 0.75 by decreasing the As flux from 1.61 × 10^−6^ to 4.20 × 10^−7^ Torr, as confirmed by EDX and PL measurements. A recent study by Koivusalo et al. [63] revealed that, As_4_ enhances vertical NWs growth, while extending the NW growth window towards lower temperatures and higher Sb fluxes. Specifically, at a relatively low temperature of 600 °C, vertical NW growth was completely suppressed using As_2_ (Figure 3d), whereas, vertical NWs growth was still realized with As_4_ (Figure 3i). The authors added that it is possible to reach higher Sb compositions by using As_4_ to extend the growth window. The relative Sb flux could be increased beyond 80% by possibly reducing the As flux, while decreasing the temperature. By exploiting the As4 related extension of the NWs growth window, Sb incorporation was enhanced with improved optical emission up to 1400 nm wavelength range.

A two-step growth temperature has also been utilized to overcome the Sb surfactant effect. Deshmukh1 et al. [88] conducted a systematic study of the Ga-assisted MBE growth of Sb-rich GaAsSb NWs on Si (111) by varying essential growth precursors of Ga, As, and Sb beam equivalent pressure (BEP). A two-step growth temperature sequence of a high-temperature growth at 620 °C followed by growth at a lower temperature of 550 °C combined with a low BEP of Ga and low As background pressure was found to be critical for achieving a well-faceted NW morphology with a low parasitic layer on the Si (111) substrate. A high Sb composition of 80 at.% was successfully obtained in as grown GaAsSb NWs. It was observed that the growth of Sb-rich, high-quality GaAsSb axial NWs can only be realized within a narrow growth space. Similarly, Ahmad et al. [33] utilized the two-step growth technique combined with a gradual manipulation of the Ga-BEP, to realize the growth of long NWs with homogeneous Sb composition up to 34 at.% and PL emission reaching 1.3 μm at room temperature. They further reported an enhanced Sb incorporation with decreasing growth temperature, which is associated with the lower volatility of Sb, (in comparison to As).

## 4. Applications of Au-Free III–As–Sb Nanowires

Monolithically integrated gold-free Ternary III–V–Sb NWs compatible with Si CMOS technology on Si have shown promise for the fabrication of novel nanodevices. Although, there are only a few reports of Au-free ternary III–V–Sb NWs optoelectronic devices, which is unsurprising given the difficulty in fabricating these NWs on Si. It is only recently that high-performance, silicon-based, optoelectronic nanodevices were developed. In this section, the optical properties of Au-free ternary III–V–Sb NWs were first presented, followed by a review of the device applications of the NWs.

### 4.1. Optical Properties of Au-Free Ternary III–As–Sb Nanowires

To successfully integrate Ternary III–V–Sb NWs with the well-established Si technology in novel nanodevices, it is crucial to realize the growth of high-quality NWs with the high optical property. The silicon-based optoelectronics device application of Sb-based NWs with extended wavelengths is highly dependent on the amount of Sb incorporated. It is therefore imperative that growth strategies are developed to increase the Sb content. However, optically efficient InAsSb NWs with a high Sb content remains a challenge, due to poor crystalline and material quality of the NWs.

**Figure 8:**
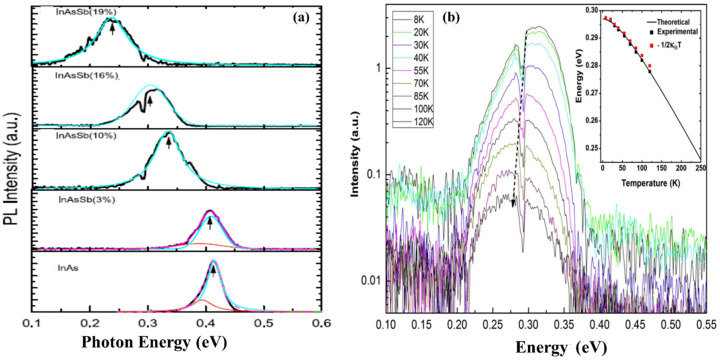
(**a**) The evolution of 10 K PL of InAs NWs and InAsSb NWs with an Sb composition of 3%, 10%, 16% and 19%. The peaks in colors show the decomposed emissions. (**b**) The photoluminescence of InAs0.84Sb0.16 NWs measured at various temperatures. The inset is the peak energy *E_p_*, the energy of *E*g–*k*_B*T*_*/*2, and the theoretical bandgap energy. Reproduced with permission from [87]; Copyright 2017, IOP publishing Ltd.

**Figure 9:**
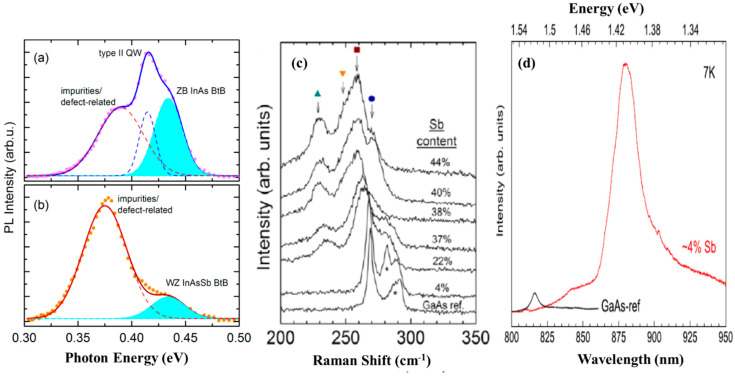
10 K PL spectra of (**a**) InAs NWs showing three emission peaks associated with impurity/defect related, type II quantum wells (QWs) formed from the ZB and WZ crystal phase mixtures and ZB InAs band to band (BtB) transitions and (**b**) InAs_0.96_Sb_0.04_ NWs which clearly exhibits a band-to-band (BtB) associated emission from WZ phase without the presence of type II QW emission, which indicates the quasi-pure WZ phase in the InAsSb NWs. Reproduced with permission from [53]; Copyright 2015, American Chemical Society. (**c**) Room-temperature Raman spectra of GaAsSb nanowires on a silicon substrate with increasing Sb content. The spectra are shifted for clarity. Alloy modes are marked by symbols and SO modes by an asterisk. (**d**) Photoluminescence (PL) spectrum of the GaAsSb NWs with the lowest Sb content in this work. The PL of GaAs reference NWs grown with the same system is also shown. Reproduced with permission from [59]; Copyright 2013, IOP publishing Ltd.

Recently, high-quality and optically efficient, pure ZB InAsSb NWs with Sb content of ~19% covering the midinfrared wavelength (3.0–5.1 μm) suitable for application in mid-wavelength infrared, silicon-based optoelectronics have been realized by MBE [87]. The room-temperature PL emission wavelength of InAs NWs was successfully broadened to 5.1 μm in the InAsSb NWs. Figure 8a shows the 10 K PL spectrum of InAsSb NWs with increasing Sb composition. The temperature-dependent PL spectra of the NWs depicted in Figure 8b clearly shows a redshift with increasing temperature up to 120 K. The optical properties of MBE grown InAsSb NW ensembles on Si has also been elucidated [53]. The low-temperature (10 K) PL spectra of the reference InAs NWs and As-grown InAsSb NWs depicted in Figure 9a shows the InAs NWs display a multipeak emissions centered at ~0.389, 0.415, and 0.434 eV and attributed to the impurity or defect-related transition, type II quantum wells (QW) related emission from the WZ/ZB mixture, and the ZB band-to-band (BtB) transition, respectively. On the other hand, the InAsSb NWs with 4% Sb clearly exhibit a WZ phase associated with BtB emission without the presence of type II QW emission, which confirms the quasi-pure WZ phase of as-grown InAs_0.96_Sb_0.04_ NWs (Figure 9b).

GaAsSb NWs holds enormous promise for optoelectronic and energy harvesting application owing to their wideband structure tunability. Consequently, it is crucial to control their composition and strain to have a better understanding of their optical and electronic properties. Raman scattering is one of the key techniques that can be used to extract vital information on the vibrational properties of materials related to the composition and strain. Alarcón-Lladó et al. [59] systematically investigated the vibrational properties of MBE grown GaAsSb NWs on Si for Sb contents in the range of 0–44%, as determined by EDX analyses. Representative raman spectra of the samples are depicted in Figure 9c. In the back-scattering configuration from a (111) face of a ZB crystal, both transverse (TO) and longitudinal optical (LO) modes are allowed. The spectrum of the GaAs NWs reference sample is dominated by two peaks at ~268.8 and 291.5 cm^−1^, associated with the TO and LO modes of binary unstrained ZB GaAs, respectively. The authors discovered that the TO intensity of the GaAs reference was about 4× larger than that of the LO mode while an intense shoulder at the low-frequency side of the LO peak was attributed to a surface optical mode, commonly observed in thin semiconductor NWs. As the Sb content was increased, the TO and LO peaks of GaAs were significantly broadened, due to an increased degree of disorderliness and were shifted to lower frequencies with increasing Sb concentration owing to the presence of the relatively heavy Sb atom in the lattice. The low-temperature PL spectra of the GaAs reference NW along with that of a GaAs_0.96_Sb_0.04_ NW taken at the same power excitation are shown in Figure 9d. As can be seen, the main PL peak of the GaAs_0.96_Sb_0.04_ NW is centered at 1.4 eV, with weaker emissions at both higher and lower energies also detected. In addition, its PL spectrum is more than 10× stronger than that of the reference GaAs NWs, which is rather dominated by the free exciton at 1.52 eV and defect-mediated exciton recombination at lower energies (around 1.5 eV). The authors believe that the significantly higher PL intensity observed in the GaAs_0.96_Sb_0.04_ NW compared to the GaAs NWs could be at least in part, due to Sb passivation. This work provides a reference for future studies of ternary NWs-based alloys and demonstrates the tunability and high material quality of gold-free ternary antimonide NWs directly grown on silicon.

### 4.2. Device Applications of Au-Free Ternary III–V–Sb Nanowires

**Figure 10:**
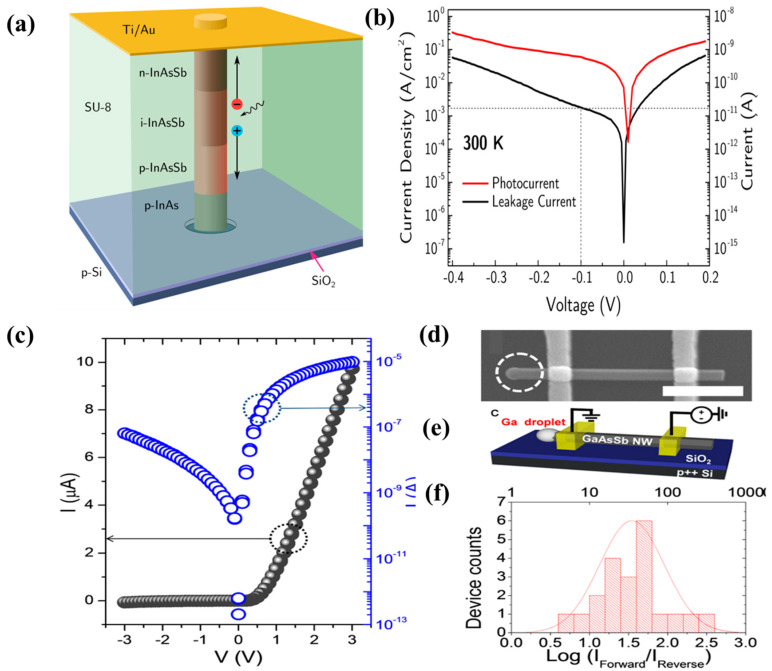
Schematic of p–i–n InAsSb nanowire photodiode. (**a**) The nanowire consists of a 200 nm Be doped InAs stub followed by 500 nm Be doped InAsSb, 500 nm undoped InAsSb, and 500 nm Te doped InAsSb. The nanowire is encapsulated by cross-linked SU-8 with Ti/Au forming the top contact. (**b**) Semilog current−voltage characteristic of InAs_0.93_Sb_0.07_ p–i–n photodiode at 300 K with a nanowire diameter of 80 nm and a length of 1.7 μm. The black and red plots show the leakage current and photocurrent density, respectively, for 200 contacted nanowires. At a typical operational bias of −0.1 V the leakage current is 18 pA, which corresponds to a leakage current density around 2 mA/cm2. For photocurrent measurements, illumination was provided by a 1.55 μm laser to which the substrate is transparent. Reproduced with permission from [44]; Copyright 2016, American Chemical Society. (**c**) Representative current–voltage (I–V) characteristics of a single GaAsSb NW device showing a rectifying behavior. (**d**) SEM image of the GaAsSb NW device with the Ga droplet marked by a dashed circle. The scale bar is 1 μm. (**e**) Schematic of the single GaAsSb NW device. The GaAsSb NW is placed on a SiO_2_/p+2-Si substrate and contacted with two identical metal electrodes. (**f**) A histogram of the current rectification ratio (IForward/Ireverse) at ±3 V for 21 different NW devices. The distribution is fitted with a Gaussian curve. Reproduced with permission from [90]; Copyright 2015, American Chemical Society.

A major step toward high-performance mid-infrared photodetectors compatible with silicon technologies enabling integration with other photonic systems was achieved in 2015 by Thompson et al. [44]. The authors fabricated InAsSb NWs photodetectors for shortwave infrared detection using axially doped p–i–n InAsSb NWs arrays grown on Si substrates by SAG. Figure 10a,b shows the schematic of the p–i–n InAsSb NW photodiode and the semi log current–voltage characteristic of the InAs_0.93_Sb_0.07_ p–i–n photodiode at 300 K with an NW diameter of 80 nm and a length of 1.7 μm. The devices exhibited a low leakage current density around 2 mA/cm^2^ and a 20% cut off of 2.3 μm at 300 K. This record low leakage current density demonstrates the suitability of III–V–Sb NWs for integration with the mature silicon technology. Robson et al. [89] demonstrated the control of NW diameter and multispectral absorption of InAsSb NWs on Si by selective-area catalyst-free MBE growth technique. Using Sb flux, the axial and radial growth rates were controllably manipulated to achieve large NWs diameters, spanning 440–520 nm, necessary for optimum IR absorption. Spectroscopy measurements reveal absorptance peaks in the short-wave IR region, due to HE11 guided-mode resonances confirmed by theoretical simulations. Due to the dependence of the HE11 resonance absorption on NW diameter, multispectral absorption was demonstrated in a single material system and a single epitaxial growth step without the need for bandgap tuning. This work demonstrated the potential of InAsSb NWs for multispectral photodetectors and sensor arrays in the short wavelength IR region.

The unidirectional propagation of carriers in a semiconductor is one of the crucial components required for applications in electronic and optoelectronic devices. Huh et al. [90] demonstrated a highly reliable and reproducible high-performance self-induced rectifying behavior in GaAsSb NWs grown by an SCG on Si substrates using MBE. Representative I–V characteristics of a single GaAsSbNW devices, and the SEM image of the device, are depicted in Figure 10c,d, respectively. Using a combination of confocal micro-Raman spectroscopy, electron microscopy, and electrical measurement, the authors demonstrated that the rectifying direction is determined by the NW growth direction and the behavior associated with the asymmetric distribution in the acceptor concentration induced by radial Sb out-diffusion during the NW growth. Figure 10e,f shows the schematic of the single GaAsSb NW device and histogram of the current rectification ratio (I_Forward_/I_Reverse_) at ±3 V for 21 different NW devices. The authors convincingly proved that GaAsSb NWs holds enormous promise as photodetectors and logic circuits, while avoiding the common complex fabrication processes.

A proper understanding of the interfacial and material properties of GaAsSb NW devices is essential for high-performance electronic and optoelectronic devices. Consequently, Huh et al. [91] investigated the electrical properties of a GaAsSb NW Schottky diode using static and low-frequency noise analysis. The authors reported that the GaAsSb NW device exhibited 1/f dependency in the noise spectrum. The Hooge’s noise parameter associated with defects and/or quality of the GaAsSb NW was found to be around 2.2 × 10^−2^, while the interface trap density of the Schottky diode was estimated to be ~2 × 10^12^ eV^−1^ cm^−2^ using a random walk noise model. These results demonstrate that the GaAsSb NW Schottky diode has better quality and interface trap density, compared to other NW based devices. The observed low-frequency noise properties could potentially provide guidance on the quality and reliability of GaAsSb NW based electronic devices, especially for photodetectors.

In 2020, the electrical probing of carrier separation in catalyst-free CBE grown InAs/InP/GaAsSb core-dual shell (CDs) NWs on Si (111) substrates were investigated by Salimian et al. [92]. A field-effect modulation of charge transport was demonstrated in the core-shell devices indicating the n- and p-type nature of the InAs core and the GaAsSb shell, respectively, which suggests that the InAs/InP/GaAsSb core-dual shell NW is suitable for the investigation of the physics of interacting electrons and holes at the nanoscale. Investigation of the influence of the InP shell epitaxially grown between the n-type core and the p-type shell of a core dual shell NW on the electronic transport revealed that the thin radial insulating barrier successfully quenched the tunnel coupling between electrons and holes located in the two axial channels. It was demonstrated that the InAs/InP/GaAsSb CDs-NW-based device has the potential to function as a multifunctional device permitting axial transport in two parallel semiconductor channels with opposite doping, as well as radial transport across the two channels, which demonstrates that the potential of the InAs/InP/GaAsSb CDS NW-based devices as a suitable platform for engineering Coulomb-coupled electron-hole systems in individual nanostructures, as well as investigation of the physics of interacting electrons and holes at the nanoscale. In the same year, the potential of GaTe for use as Te-dopant in GaAsSb NWs was successfully demonstrated by Devkota et al. [93] as confirmed by enhanced PL emission, and the increased photocurrent with the significant reduction in turn-on voltage. The suitability of Te as an n-type dopant is associated with its relative ease of high-level doping at a lower temperature, high solubility in the Ga droplet, and high incorporation efficiency and low diffusion coefficient [94]. The fabricated n-GaAsSb NWs ensemble Photodetectors demonstrated a broad spectral response with a long cut off wavelength of 1.2 μm and improved responsivity of 580–620 A/W with detectivity in the range of 1.2 × 10^12^–3.8 × 10^12^ Jones. This demonstrates the enormous potential of GaTe as a suitable n-type dopant, as well as the application of doped GaAsSb NWs for high-performance near-infrared photodetection.

## 5. Conclusions

Recent advances towards the monolithic integration of III–V–Sb nanowires on Si has been explicated. This review presents the recent progress made in the Au-free, direct epitaxial growth of high-quality, III–V–Sb NWs on silicon, while highlighting various growth strategies for mitigating Sb surfactant effect and enhancing Sb incorporation. Successful demonstrations of high-performance optoelectronic devices compatible with silicon technologies are also summarized. This demonstrates the enormous potential of gold-free Ternary III–V–Sb NWs for optoelectronic device applications. With further optimization in NWs growth strategies and improvement in device design, it is hoped that the device performance would be significantly improved. It is believed that this review would serve as a blueprint for exploiting the enormous potential of gold-free Ternary III–V–Sb NWs in future device integration on silicon.

## Figures and Tables

**Table 1:** Influence of Sb composition (*x*) on III–As–Sb Nanowires Morphology.

S/N	Material	Growth Tech.	Growth Strategy	Time (min)	Temp (°C)	As Flux (Torr)	Vapor Phase (%)	Sb Flux *	L (μm)	D (nm)	(*x*) (at.%)	Ref.	Remarks
1	InAsSb	MBE	SC	20	420–470		0.79	5.7 × 10^−8^	0.87	109	4.3	[52]	
							4.95	3.75 × 10^−7^	0.83	115	4.5		
2	InAsSb	MBE	SC	120	420–470		2.93	5.7 × 10^−8^–3.75 × 10^−7^	0.7	155	10.2	[32]	2D film obtained for *x* = 14.5%
3	InAsSb	MOVPE	SS	2	510		0.2	0.8 × 10^–5^	0.28	~50	~08	[36]	
							0.4		0.17	~90	~14		
							0.6		0.12	~240	~23		
							0.8		0.00	~480	~43		
4	GaAsSb	MBE	SC	20	620		-	-	5.7	~ 86	2.8	[61]	
									5.4	~100	7.5		
									3.5	~110	11		
									2.5	125	16		
5	GaAsSb	MBE	SC	35	625	2.5 × 10^−6^	-	2 × 10^−7^	3.00	~250	06	[46]	
				35	625	2.5 × 10^−6^	-	4 × 10^−7^	2.65	~280	15		
				35	625	2.5 × 10^−6^	-	6 × 10^−7^	2.45	~315	16		
				35	625	2.5 × 10^−6^	-	8 × 10^−7^	2.05	~335	19		
6	GaAsSb	MBE	SC		590	2.25 × 10^−6^		4.50 × 10^−7^	** 79	** 75	18	[60]	2D film obtained for *x* = 75%
						2.25 × 10^−6^		6.75 × 10^−7^	** 71	** 140	27		
						2.25 × 10^−6^		1.43 × 10^−6^	** 22	** 175	35		
						1.61 × 10^−6^		4.50 × 10^−7^	** 74	** 130	30		
						9.75 × 10^−7^		4.50 × 10^−7^	** 49	** 192	60		

**KEY:** SC = Self-catalyzed, SS = Self-seeded. ** Growth rate in nm/min, * Sb flux is in (Torr) for MBE and mol/min for MOVPE grown NWs.
